# Computational Drug Repurposing Approach to Identify Novel Inhibitors of ILK Protein for Treatment of Esophageal Squamous Cell Carcinoma

**DOI:** 10.1155/2022/3658334

**Published:** 2022-12-29

**Authors:** Juan Liu, Xiaoli Ma, Leiyu Cao, Yu Wei, Yan Gao, Chengcheng Qu, Nuersimanguli Maimaitiming, Li Zhang

**Affiliations:** Department of General Medicine of Healthy Care Center for Cadres, The First Affiliated Hospital of Xinjiang Medical University, Urumqi, Xinjiang Uygur Autonomous Region, China

## Abstract

**Purpose:**

Esophageal squamous cell cancer (ESCC) is a deadly malignant tumor characterized by an overall 5-year survival rate below 20%, with China accounting for approximately 50% of all cases worldwide. Our previous studies have demonstrated that high integrin-linked kinase (ILK) expression plays a key role in development and progression of ESCC both *in vitro* and *in vivo*. Here, we employed the drug repurposing approach to identify a novel FDA-approved anticancer inhibitor against ILK-induced tumorigenesis and progression.

**Methods:**

We screened the ZINC15 database and predicted the molecular docking ability among FDA-approved and publicly available drugs to ILK and then performed computational docking and visual inspection analyses of the top 10 ranked drugs. Two computer-based virtual screened drugs were evaluated *in vitro* for their ability to directly bind purified ILK by surface plasmon resonance. Cytotoxicity of the two candidate drugs was validated *in vitro* using CCK-8 and LDH assays.

**Results:**

We initially selected the top 10 compounds, based on their minimum binding energy to the ILK crystal, after molecular docking and subjected them to further screening. Taking the binding energy of −10 kcal/mol as the threshold, we selected two drugs, namely, nilotinib and teniposide, for the wet-lab experiment. Surface plasmon resonance (SPR) revealed that nilotinib and teniposide had equilibrium dissociation constant (KD) values of 6.410*E* − 6 and 1.793*E* − 6, respectively, which were lower than 2.643*E* − 6 observed in ILK-IN-3 used as the positive control. The IC50 values for nilotinib and teniposide in ESCC cell lines were 40 *μ*M and 200–400 nM, respectively. Results of the CCK-8 assay demonstrated that both nilotinib and teniposide significantly inhibited proliferation of cells (*P* < 0.01). LDH results revealed that both drugs significantly suppressed the rate of cell death (*P* < 0.01).

**Conclusion:**

The drug repositioning procedure can effectively identify new therapeutic tools for ESCC. Our findings suggest that nilotinib and teniposide are efficacious inhibitors of ILK and thus have potential to target ILK-mediated signaling pathways for management of ESCC.

## 1. Background Information

Esophageal cancer is one of the most common malignant tumors that threaten human health and life. The disease is ranked 7th and 6th with regards to morbidity and mortality among global malignant tumors, respectively. In China, esophageal cancer accounts for about 43.0% and 37.0% of all global new cases and deaths worldwide, respectively, with esophageal squamous cell cancer (ESCC) shown to be the main subtype [1, 2]. ESCC has a 5-year survival rate of about 30%, due to its atypical early symptoms, high malignancy, aggressiveness, and more limited treatment options [3].

Despite extraordinary advances in the cancer biology field, progress in the area of drug research and development (R&D) remains slower than expected [4]. A promising solution to the considerable drug development challenges of novel compounds is drug repurposing (also called drug repositioning, reprofiling or retasking), a strategy for identifying new clinical indications for approved or investigational drugs [5]. Approved drugs have undergone all phases of clinical trials in order to reach the market and thus have known and accepted safety data, which substantially reduce the R&D risk, time, and cost [6]. Sildenafil, a phosphodiesterase type 5 (PDE5) inhibitor, is a successful drug repurposing example. Sildenafil was originally developed to treat hypertension but was later approved by the FDA for the treatment of erectile dysfunction and pulmonary hypertension for the same [7, 8].

The participation of computer-aided drug design (CADD) can minimize the failure rate of research and development and further consolidate the advantages of drug repositioning, which includes two major types of drug design techniques: ligand-based drug design (LBDD) and structure-based drug design (SBDD) [9]. As one of the most commonly used in in silico approaches, SBDD utilizes the structural information from a 3D protein structure to predict macromolecular binding sites and ligand affinity [10]. Molecular docking is a typical SBDD method used to evaluate binding affinities between proteins and ligands [11]. For example, with performing molecular fit computations on 3,671 FDA-approved drugs across 2,335 human protein crystal structures, Dakshanamurthy et al. screened that mebendazole, an antiparasitic drug, has the structural potential to inhibit the angiogenic medium vascular endothelial growth factor receptor 2 (VEGFR2), a mediator of angiogenesis, which was also confirmed by experiments [12].

Integrin-linked kinase (ILK), an intracellular protein kinase, is not only involved in several signal transduction processes but also regulates the cell cycle, migration, and growth among other biological processes [13]. Previous studies have shown that high ILK expression mediates regulation of cell proliferation, migration, and differentiation in a variety of tumors and also affects therapeutic effects of tumors [14–16]. Meanwhile, we found that ILK was highly expressed in ESCC, while its interference using the lentivirus markedly inhibited occurrence and development of ESCCs [17]. This project aims to screen some small molecules to inhibit ILK with high selectivity and activity that have become drugs through computer-aided drug research and to conduct primary screening and validation of their pharmacological activities so as to provide experimental and theoretical support for the research of novel anticancer-led compounds.

## 2. Materials and Methods

### 2.1. Cell Cultures and Reagents

Human ESCC cell lines KYSE150 and TE-1 were purchased from the Cell Bank at the Chinese Academy of Science (Shanghai, China). Cells were cultured in RPMI1640 medium, supplemented with 10% fetal bovine serum (FBS) (Gibco, Rockville, MD, USA) and 1% penicillin-streptomycin (NCM Biotech, Soochow, China). All cell cultures were incubated at 37°C and 5% CO_2_ in a humidified atmosphere. Nilotinib was purchased from MedChemExpress Co., Ltd. (Shanghai, China), while teniposide and ILK-IN-3 were acquired from Target Molecule Corp. (Shanghai, China). All compounds were dissolved in DMSO (MCE, Shanghai, China) before use.

### 2.2. Virtual Screening

First, we obtained a three-dimensional structure of ILK protein from the ILK/alpha-parvin core complex crystal structure downloaded from the PDB database [18] and then extracted the ILK protein (A chain) using the PyMOL tool. Subsequently, the potential binding pockets of ILK protein were determined by using the DoGSiteScorer online tool [19], which was developed to predict the binding pockets and rank them based on the size, surface area, and druggability score. The chosen binding pocket was further validated by fpocket [20] and CASTp [21] tools. Next, we obtained the list of 1615 FDA-approved drugs from the ZINC database [22] for virtual screening. These drugs were converted to PDB formats using MGLTools and then subjected to molecular docking using the AutoDock Vina docking program [23]. We ranked the drugs by their binding affinities when bound to binding pockets. Finally, we selected 2 drugs with minimum free energy for wet-lab validation.

### 2.3. *In Vitro* Assessment of Direct Drug Binding to ILK

SPR experiments were carried out using a Biacore S200 instrument (Biacore, GE Healthcare, Boston, MA, USA) at 25°C. Summarily, ILK (ligand) was first immobilized to the activated CM5 sensor chip by amine coupling with pH 5.0 and sodium acetate concentration of 46 *μ*g/mL. The nonreaction group was then blocked by injection of ethanolamine hydrochloride 1 M (35 *μ*L). The drug (analyte) was dissolved in 100% DMSO (to an initial concentration of 10 mM) and diluted to 200 *μ*M (5% final DMSO concentration) with 20 mM HEPES, 150 mM NaCl, and 0.005% surfactant P20 buffer (HBSP). It was further diluted with HBSP + 5% DMSO (HBSP5%D). We then confirmed concentrations of compounds based on the solubility and the test results from sensor chips of samples to be measured. An increase in the RU value from the baseline indicates that formation is complex and that the plateau region represents a steady-state phase of interaction (RUeq), whereas a decrease in RU after 100 s indicates separation of the analyte from immobilized ILK after injection of HBSP5%D buffer. Finally, we performed sensorgram analysis using the Biacore S200 evaluation software.

### 2.4. Evaluation of Anticancer Activity of the Two Screening Compounds

#### 2.4.1. CCK-8 Assay

KYSE150 and TE-1 cells were, respectively, seeded into 96-well culture plates (5 × 10^4^ cells per well) and incubated for 24 h at 37°C, 5% CO_2_, and 100 *μ*L of different concentrations of the candidate drugs was prepared and added to 1640 medium, followed by 48h incubation. The medium was refreshed; then, 100 *μ*L of the medium with 10% CCK-8 was added to each test well, followed by 2 h of incubation at 37°C. A microplate reader (BioTek Instruments, Inc., Winooski, VT, USA) was used to measure OD at 450 nm in each well, and changes in cell proliferation ability were detected.

#### 2.4.2. LDH Assay

KYSE150 and TE-1 cells were, respectively, seeded into 96-well plates (5 × 10^4^ cells/well) and incubated for 24 h at 37°C, 5% CO_2_. 150 *μ*L of different concentrations of the candidate drugs was added to 1640 medium and incubated for 48 h, followed by addition of 15 *μ*L of LDH release reagent with maximum enzyme activity. The contents were incubated for 1 h and centrifuged; then, 100 *μ*L of each of the supernatants was added into the corresponding well of a 96-well plate. A microplate reader was used to measure OD in each test well at 490 nm, and changes in cytotoxicity were detected.

## 3. Results

### 3.1. Identification of Potential ILK Binding Drugs by Virtual Screening

#### 3.1.1. ILK Protein and the 3D Crystal Structure

**Figure 1:**
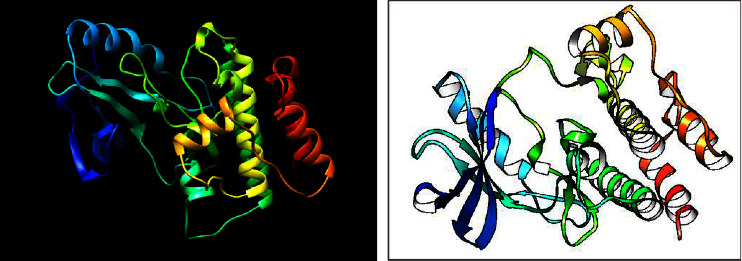
3D structure of the ILK protein.

The three-dimensional structure of the ILK protein was obtained from the ILK/alpha-parvin core complex crystal structure in the PDB database (3KMU) according to ILK protein sequence (supplementary file). Next, the ILK protein (A chain) was extracted using PyMOL, and its three-dimensional structure is shown in Figure 1.

#### 3.1.2. Prediction of Binding Pockets

We utilized the online tool DoGSiteScorer for prediction and description of potential binding pockets of the ILK protein. The top 9 predicted binding pockets are listed in Table 1. We only considered the first binding pocket during molecular docking, due to the fact that its contact surface area was much larger than that of others. Meanwhile, we used two other popular tools, fpocket and CASTp, to predict the binding pocket and found similar results.

#### 3.1.3. Molecular Docking and Screening Results

We screened the FDA-approved drug library, downloaded from the ZINC 15 database, for potential hits during our virtual screening. The library contains 1615 FDA-approved drugs. The drugs were retrieved in the MOL2 format and then converted to the .pdbqt format using MGLTools. During transformation, both Gasteiger charges and polar hydrogen atoms were added, and drugs were prepared for docking.

**Figure 2:**
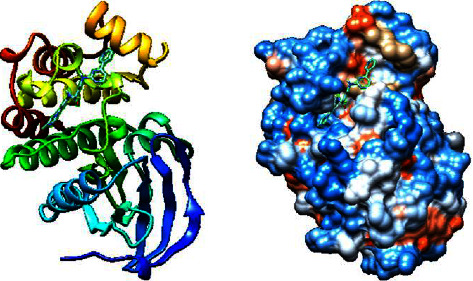
3D illustration of molecular docking of nilotinib molecules with ILK protein.

**Figure 3:**
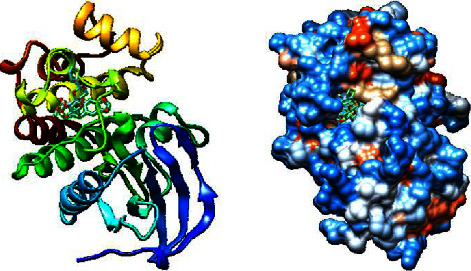
3D illustration of the molecular docking of teniposide and ILK protein.

Next, we utilized AutoDock Vina tools to dock FDA-approved drugs and ILK protein using the following molecular docking parameters: a binding site area of (28*∗*30*∗*32) angstrom with coordinates of (*X*: 6.292, *Y*: 18.998, and *Z*: 12.265). The output format of the data was sorted by the minimum binding energy of these drugs to the ILK crystal. Next, we selected the top 10 hits from the molecular docking for further screening, as shown in Table 2. Taking the binding energy of −10 kcal/mol as the threshold, two drugs, namely, nilotinib (ZINC6716957) and teniposide (ZINC4099009), were selected for subsequent wet-lab experiments. For each drug, the ligand-target complex with the least binding energy pose is illustrated in Figure 2 and Figure 3, while information for their chemical formulas is listed in Table 3.

#### 3.1.4. Assessment of Direct Drug Binding to ILK *In Vitro*

**Figure 4:**
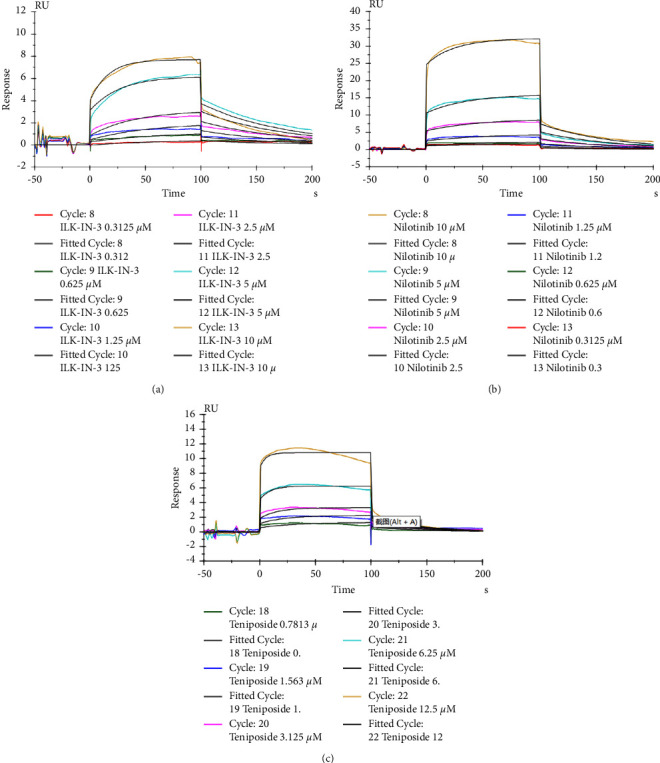
Sensorgram plots for the compounds selected by virtual screening and computational docking ((b) nilotinib; (c) teniposide) and of ILK-IN-3 (a) as the control.

To validate the virtual screening results, we carried out SPR *in vitro* to evaluate the biomolecular interaction of drugs. Summarily, the recombinant ILK protein was immobilized on an activated CM5 chip with the pH value of the most suitable coupling reaction. The results of the ability of ILK protein to bind the two drugs at various concentrations are shown in Figure 4. ILK-IN-3 was included as a positive control [24]. The SPR results showed that the top 2 drugs with strong affinities predicted by virtual screening could indeed bind to ILK. Biacore S200 software revealed that the drugs' dissociation constant (KD) values were in the micromolar range and were comparable to those of the control (Table 4). Interestingly, teniposide (KD = 1.793 *μ*M) had a higher affinity for ILK than for ILK-IN-3 (KD = 2.643 *μ*M).

### 3.2. Evaluation of Anticancer Activity in the Two Compounds

**Figure 5:**
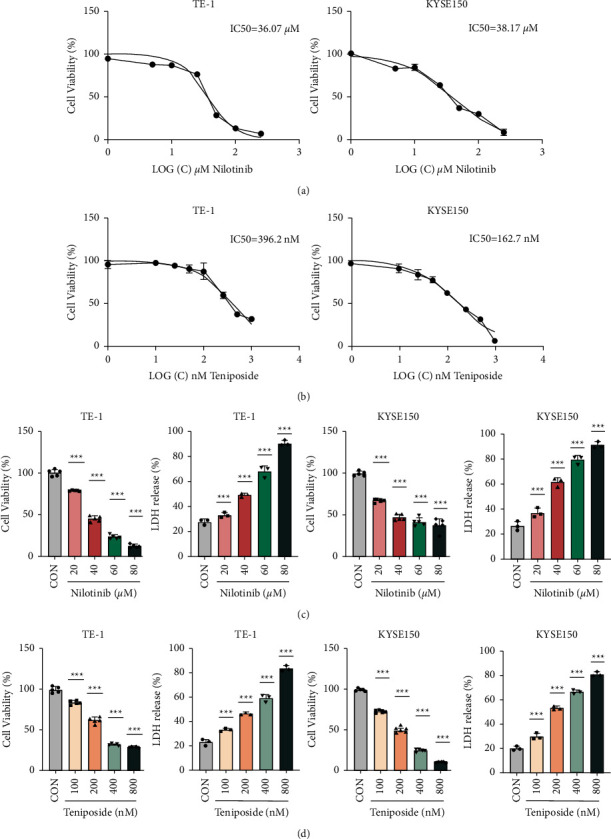
Evaluation of the anticancer activity of two screening compounds. (a, b) IC50 values of nilotinib and teniposide in ESCC cells. TE-1 and KYSE150 cells were treated with different concentrations of two drugs for 48 hours. The cell viability was assessed by CCK8 assay, and IC50 values were calculated. (c, d) Cell viability and LDH release of ESCC cells (TE-1 and KYSE150) detected using the CCK8 and LDH assay after treatment of nilotinib and teniposide conditioned medium or control medium (^∗∗∗^*p* < 0.001).

Next, we used CCK-8 and LDH release assays to determine the function of the two compounds in proliferation and death of ESCC cells. To this end, we targeted KYSE-150 and TE-1 human esophagus squamous cancer cells, in which ILK was expressed at relatively higher levels than other ESCC cells according to our previous studies [17]. Notably, nilotinib had an IC50 value of 40 *μ*M, while the IC50 value of teniposide ranged from 162.7 to 396.2 nM in these ESCC cell lines (Figures 5(a) and 5(b)). This suggested that teniposide has a relatively higher cytotoxic effect on ESCC cells than on nilotinib, consistent with the SPR results. Moreover, an increase in drug concentration was associated with a decrease in viability and an increase in death of ESCC cells (Figures 5(c) and 5(d)).

## 4. Discussion

The research on ESCC therapy progress is slow; therefore, there is an urgent need to identify effective antitumor therapeutic drug targets. Generally, drug development is a difficult task that takes a long period of time and is accompanied by high costs and high risk as well as low payoffs. In contrast, drug repurposing, which is an approach to discover a new effect for an already approved drug in novel disease types, offers a higher success rate, lowers the risk ratio, and shortens the time taken before clinical use [6]. Notably, researchers are currently employing virtual screening (VS) based on target structures as an effective method for new drug discovery. This approach not only allows for rapid screening of a large number of compounds in drugs within a short period of time but is also a novel way to discover new drugs, as evidenced by recent successes [25, 26]. Since most small molecules may have multiple molecular targets, the discovery of new therapeutic targets for a drug is essential for new indications [27].

ILK was originally thought to be a serine/threonine protein kinase that interacts with the cytoplasmic domain of *β*-1 integrin and mediates the connection between cells and the extracellular matrix. Functionally, it affects the transmission of extracellular signals and also plays a central role in regulating fundamental processes, such as cell morphology, motility, growth, survival, differentiation, and gene expression [13]. To date, the exact molecular mechanism underlying ILK signal transduction has been quite controversial [28]. However, a recent study showed that ILK is a pseudokinase [29]. Subsequently, numerous studies have revealed that ILK plays a key role in epithelial-mesenchymal transition (EMT), invasion, and angiogenesis, suggesting that it could be an attractive target for tumor therapy [30–32]. In fact, some studies have shown that ILK is not only upregulated in a variety of malignancies but is also associated with a poor patient prognosis. At the same time, our research group previously demonstrated that interfering with ILK can significantly inhibit proliferation, invasion, and migration of ESCC cells and also improve patient prognosis [17, 33]. ILK, as a therapeutic target, has attracted a lot of research attention. For instance, some researchers have designed small-molecule compounds targeting ILK to inhibit tumor growth, although these studies have neither clearly described whether these compounds directly bind to ILK nor demonstrated their specificity, which necessitates further exploration before their clinical application [24, 34–36].

In the present study, we screened two known drug compounds with strong ILK binding affinity, nilotinib and teniposide, from the ZINC15 drug database, containing 1615 FDA-approved drugs, using the VS approach based on the ILK protein structure. In addition, we validated purified ILK proteins using molecular biology techniques and measured the inhibitory effects of these compounds on ILK with a view of verifying reliability of the computerized virtual screening results. Our results showed that both nilotinib and teniposide had a stronger binding affinity to ILK than the positive reference compound ILK-IN-3, a phenomenon that initially demonstrated the feasibility of the protein structure-based drug virtual screening method. Subsequently, we evaluated the antitumor activity of the two inhibitors on ESCC TE-1 and KYSE150 cells and found that both inhibitors significantly promoted cell death and inhibited proliferation, consistent with drug virtual screening results.

These results suggest that the observed antitumor effects of nilotinib and teniposide on ESCC are partially related to ILK's binding ability coupled with their inhibitory effects. Nilotinib, which belongs to the second-generation tyrosine kinase inhibitor (TKI), has been used as an effective therapy in clinical treatment of chronic myeloid leukemia (CML) that is intolerant or resistant to imatinib (Gleevec) [37]. On the other hand, teniposide, a chemotherapeutic agent that targets DNA topoisomerase II, has been primarily used for treatment of acute lymphoblastic leukemia, lymphoma, and brain cancer in children [38]. However, the drug's efficacy has also been studied in other different types of solid tumors [39–41], with the resultant data suggesting that it may inhibit tumor growth through other targets. This also suggests that the drug might have multiple molecular targets, and the discovery of new therapeutic targets for the drug is essential to broaden new indications. The results of the present study, albeit preliminary, demonstrated that the two drugs have antitumor activity. In the future, we intend to validate the observed antitumor effects and underlying mechanisms of the screened drugs using *in vitro* and *in vivo* experiments.

## 5. Conclusion

There is a need to continually screen for ILK inhibitors to identify broad antitumor activity and high selectivity because of the problems of selectivity and specificity in the current research on ILK inhibitors. In this study, we employed VS, based on the ILK protein structure, to find out two potential antiesophageal squamous cell carcinoma drugs, which are nilotinib and teniposide, and verified the binding ability of ILK protein with two compounds by surface plasmon resonance (SPR). In addition, we validated the antitumor activity of the two drugs in esophageal squamous cell carcinoma cell lines. All the above results confirmed that both nilotinib and teniposide are effective and selective ILK inhibitors, and this role merits further investigation.

## Figures and Tables

**Table 1:** Information of the top 9 predicted binding pockets.

No. pocket	Volume Å³	Surface Å²	Drug score	Simple score P_1
P_1	1790.91	2139.69	0.81	0.68
P_2	494.21	564.73	0.72	0.28
P_3	492.67	762.3	0.7	0.28
P_4	378.3	674.96	0.71	0.19
P_5	355.52	753.96	0.52	0.26
P_6	242.37	426.74	0.43	0.1
P_7	148.54	274.23	0.31	0
P_8	142.34	450.42	0.24	0
P_9	114.18	263.03	0.26	0

**Table 2:** The top 10 drugs with minimum binding energy to the ILK crystal.

No.	Generic name	Molecular formula	Binding energy (kcal/mol)
d1	Nilotinib	C_28_H_22_F_3_N_7O_	−10.5
d2	Teniposide	C_32_H_32_O_13_S	−10.3
d3	Doxycycline	C_22_H_24_N_2_O_8_	−9.9
d4	Celsentri	C_29_H_41_F_2_N_5_O	−9.8
d5	Saquinavir	C_38_H_50_N_6_O_5_	−9.8
d6	Lumacaftor	C_24_H_18_F_2_N_2_O_5_	−9.7
d7	Rolapitant	C_25_H_26_F_6_N_2_O_2_	−9.7
d8	SQV	C_38_H_50_N_6_O_5_	−9.7
d9	Netupitant	C_30_H_32_F_6_N_4_O	−9.7
d10	Raltegravir	C_20_H_21_FN_6_O_5_	−9.7

**Table 3:** Chemical structure for the screened compounds.

Drug name	InChI key	SMILES	2D structure
ZINC ID			
Nilotinib	HHZIURLSWUIHRB-UHFFFAOYSA-N	Cc1cn(-c2cc(NC(=O)c3ccc(C)c(Nc4nccc(-c5cccnc5)n4)c3)cc(C(F) (F)F)c2)cn1	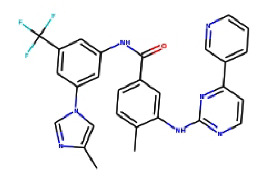
ZINC6716957			
Teniposide	NRUKOCRGYNPUPR-QBPJDGROSA-N	COc1cc([C@@H]2c3cc4c(cc3[C@@H](O[C@@H]3O[C@@H]5CO[C@@H](c6cccs6)O[C@H]5[C@H](O)[C@H]3O)[C@H]3COC(=O)[C@H]23)OCO4)cc(OC)c1O	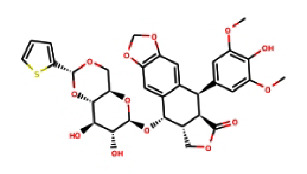
ZINC4099009			

**Table 4:** ILK affinity in selected compounds and ILK-IN-3 (control) measured by SPR experiments.

Receptor	Ligand	KD (M)	*R *_max_ (RU)
ILK protein	ILK-IN-3 (control)	2.64*E* − 06	4.724
	Nilotinib	6.41*E* − 06	12.52
	Teniposide	1.79*E* − 06	2.386

## Data Availability

The datasets used to support the findings of our study can be made available from the corresponding author on reasonable request.

## Supplementary Materials

Supplementary Materials The supplementary material contains the protein sequence of ILK.
