# Supplementary material for: Computational Drug Repurposing Approach to Identify Novel Inhibitors of ILK Protein for Treatment of Esophageal Squamous Cell Carcinoma
Source: J Oncol. 2022 Dec 29;2022:3658334. doi: 10.1155/2022/3658334 (PMC9815933; doi:10.1155/2022/3658334)
Supplement: Supplementary Materials — The supplementary material contains the protein sequence of ILK. [file 3658334.f1.docx]

Result

**1.ILK protein and 3D crystal structure**

The protein sequence of ILK is as below:

>3KMU_1|Chain A|Integrin-linked kinase|Homo sapiens (9606)

MNKHSGIDFKQLNFLTKLNENHSGELWKGRWQGNDIVVKVLKVRDWSTRKSRDFNEECPRLRIFSHPNVLPVLGACQSPPAPHPTLITHWMPYGSLYNVLHEGTNFVVDQSQAVKFALDMARGMAFLHTLEPLIPRHALNSRSVMIDEDMTARISMADVKFSFQSPGRMYAPAWVAPEALQKKPEDTNRRSADMWSFAVLLWELVTREVPFADLSNMEIGMKVALEGLRPTIPPGISPHVSKLMKICMNEDPAKRPKFDMIVPILEKMQDK
